# Face-to-face versus 360° VR video: a comparative study of two teaching methods in nursing education

**DOI:** 10.1186/s12912-024-01866-4

**Published:** 2024-03-25

**Authors:** Abdulfatai Olamilekan Babaita, Mayumi Kako, Chie Teramoto, Miho Okamoto, Yoko Hayashi, Shinichiro Ohshimo, Takuma Sadamori, Minoru Hattori, Michiko Moriyama

**Affiliations:** 1 Graduate School of Biomedical and Health Sciences, Program of Health Sciences, Hiroshima University, Hiroshima, Japan; 2 Division of Nursing Science, Graduate School of Biomedical and Health Sciences, Hiroshima University, Kasumi 1-2-3 Minami-ku, Hiroshima, 734-8553 Japan; 3 Intensive Care Unit, Hiroshima University Hospital, Hiroshima, Japan; 4 Department of Emergency and Critical Care Medicine, Graduate School of Biomedical and Health Sciences, Advanced Emergency and Critical Care Center, Hiroshima University, Hiroshima University Hospital, Hiroshima, Japan; 5 Center for Medical Education, Faculty of Medicine, Hiroshima University, Hiroshima, Japan

**Keywords:** 360º virtual reality, VR sickness symptoms, Closed tracheal suctioning, Psychomotor skills

## Abstract

**Background:**

The practical sessions during skills laboratory simulation or clinical simulation are cores of nursing education. For this, different modalities have been devised to facilitate psychomotor skills learning. One of the commonly used educational material or instructional method to supplement skills learning across various disciplines is video-based teaching method. The opportunities of traditional two-dimensional video might be limitless and maximized with 360º virtual reality (VR) video, which offers immersive experience. This study incorporates 360º VR video into skills laboratory training as an alternative approach to face-to-face procedure demonstration.

**Methods:**

An open-label, parallel (1:1), randomized controlled trial study was conducted among third-year undergraduate nursing students at Hiroshima University, Japan. The nursing students were block-randomized into 360º VR video and face-to-face demonstration group. After a 3-hour theoretical class of patient management on ventilator and closed-suction principles of mechanically ventilated patients in an Intensive Care Unit focused class, the 360º VR group watched the 360º VR video of closed tracheal suction (including oral) using the head-mounted display of Meta Quest 2 individually, while the face-to-face group attended the instructor’s demonstration. A week after the skills laboratory, the students’ psychomotor skills, knowledge, satisfaction, confidence were evaluated; the 360º VR video group’s perception was explored; Wilcoxon rank-sum test was used to compare the two groups.

**Results:**

A total of 57 students were analyzed; 27 students in the 360º VR video group and 30 students in face-to-face group. There were no statistically significant differences between both groups in skills, knowledge, and confidence. However, the face-to-face group had higher satisfaction level than the 360º VR group; this difference was statistically significant. In the 360º VR video group, 62% agreed that VR makes learning more interesting; more than half of students (62.5%) experienced VR sickness symptoms, and “feeling of drunk” is the highest. The students appreciated the ready to use, immersiveness, and realism; however, symptoms and discomfort, burdensomeness, and production limitations were improvements recommended.

**Conclusion:**

Although face-to-face demonstration is the established method of teaching psychomotor skills to nursing students, the use of 360º VR video could achieve similar learning effect as an alternative approach.

**Supplementary Information:**

The online version contains supplementary material available at 10.1186/s12912-024-01866-4.

## Background

Clinical training is an indispensable foundation in nursing education; in principle, it is the pathway to prepare nursing students, ensure competence, and achieve patient`s safety in the healthcare system. The pathway to ensuring competent nurse is the translation of theoretical knowledge to practical knowledge [1, 2]; this is enabled through skills laboratory. The practical sessions during skills laboratory simulation or clinical simulation are cores of nursing education. For this, different modalities have been devised to facilitate psychomotor skills learning. However, despite the adoption of strategies to ensure the application of theory into practice, nursing education still experiences nursing students and newly graduates with deficient practical skills [3–5].

One of the commonly used educational material or instructional method to supplement skills learning across various disciplines is video-based teaching method [6–10]. Medical students source educational videos to learn clinical skills [11], and 90% of medical students reported using videos to learn procedures [12]. Moreover, the use of educational technology is part of nursing education and nurses are forerunners [13] It is argued that learning through image is relatively experiencing the real situation or an experiential process [14]. The use of educational videos in teaching positively affects the learning process [13, 15], and has shown to enhance performance [13, 16], significantly reduce study time compared to text-based material [16], and improve confidence in performing some procedures [17].

The opportunities of traditional two-dimensional (2D) video might be limitless and maximized with 360º virtual reality (VR) video, which offers immersive experience. 360º VR video employ real-world images captured with an omnidirectional camera or multiple cameras simultaneously to create an immersive environment [18]. The term VR and 360º VR video are used interchangeably; although VR is generated by using computer graphics, 360º VR video is created from real-world images [18, 19]. It is noteworthy that the defining factor of a VR system in research reviews is the VR technology rather than the level of interactivity. The undivided attention offered by 360º positively influences conceptual and spatial learning [20]. The 360º video with head-mounted display (HMD) might provide an edge over 2D videos where environmental distractions are in view.

The potential benefits of 360º VR video on learning outcomes [21], and suitability for action-oriented activities requiring visual details, which is infeasible in a traditional 2D Video [22] has been demonstrated in research. This immersive and involvement opportunity in 360º VR video has raised a debate on its use in retention of information and enhancement of learning over traditional 2D video. Harrington et. al [23] reported 65% of students preferred 360º Video over 2D; the 360º VR video group demonstrated significant higher engagement and no difference in information retention. Contrarily, Rupp et al [24] found the overwhelming feeling of presence contributed to less information recall. It is well established that 360º VR video improves student learning performance [25–27]. A systematic review on 360º VR video technology by Baysan et al [19], which included majority of non-interactive 360º video systems, concluded that the use is convenient and effective for nursing education. For this, robust research is essential as disparities exist between studies.

In Japan, research interest in VR and using VR in nursing education is increasing. However, to the best of our knowledge, the adoption of VR in nursing education is not widespread in any country. In nursing skills laboratory, procedures are demonstrated to students by nursing instructors of the intended procedure; a web video is provided to complement for future reference. The instructors deliver the procedure to the total number of students at once; this crowding could hamper the ease of understanding and better visualization. It has been reported in research that a video-based group perceived the teaching method to facilitate ease and better understanding than live demonstration [28, 29]. Closed tracheal suction is one of the important nursing procedures in Intensive Care Unit (ICU) and involves action-oriented activities requiring visual details. If this demonstration is captured in immersive 360º VR, it could offer an individualized experience, be reused by students without the need for web video as supplement and reduce faculty dependence in future demonstration of the procedure. Moreover, video-based teaching is a self-directed learning approach and could reduce the number of instructors needed to conduct hands-on practice in nursing skills laboratory; teachers’ dependence of students is one of the negatives of live demonstration [30]. Therefore, this study incorporates 360º VR video into skills laboratory training as an alternative approach to face-to-face procedure demonstration. The purpose of this study was to examine the effects of 360º VR video and face-to-face teaching method in learning closed tracheal suction (including oral suction). We hypothesized that (1) nursing students who learned the procedure with 360º VR video would have higher skill performance scores than students who received the face-to-face demonstration, (2) the 360º VR video group would have better theoretical knowledge than the face-to-face group, (3) the nursing students who watched the 360º VR video would self-report higher satisfaction and confidence level than the students who received face-to-face demonstration, and (4) the 360º VR video would have considerably good perception of the use of the technology.

## Method

### Study design and participants

An open-label, parallel (1:1), randomized controlled trial design was conducted among undergraduate nursing students at Hiroshima University, Japan. Participants were third-year nursing students enrolled in the Practicum in Adult Nursing in 2023.

In the third year, students study each nursing science area after completing basic nursing subjects; they study theory and skills in parallel, and after completion, they go on to clinical practice.

### Data collection procedure

Since this study was implemented in the regular class, the data for this analysis was obtained after the class completion as an opt-in basis. Prior to commencement of the specified class explained below, students were informed of the purpose of this experiment, procedure, voluntarily of participation, no disadvantages of withdrawal and/or no participation, and the secondary use of the data. Then, students submitted the written consent form for providing their data submitted in the class to the researcher who was not involved in the course.

After a 3-hour theoretical class of patient management on ventilator and closed-suction principles of mechanically ventilated patients in an ICU focused class, a seven-question knowledge pretest was conducted for all the available nursing students enrolled in the course; a total of 62 students completed the pretest for randomization.

### Randomization and allocation

**Fig. 1:**
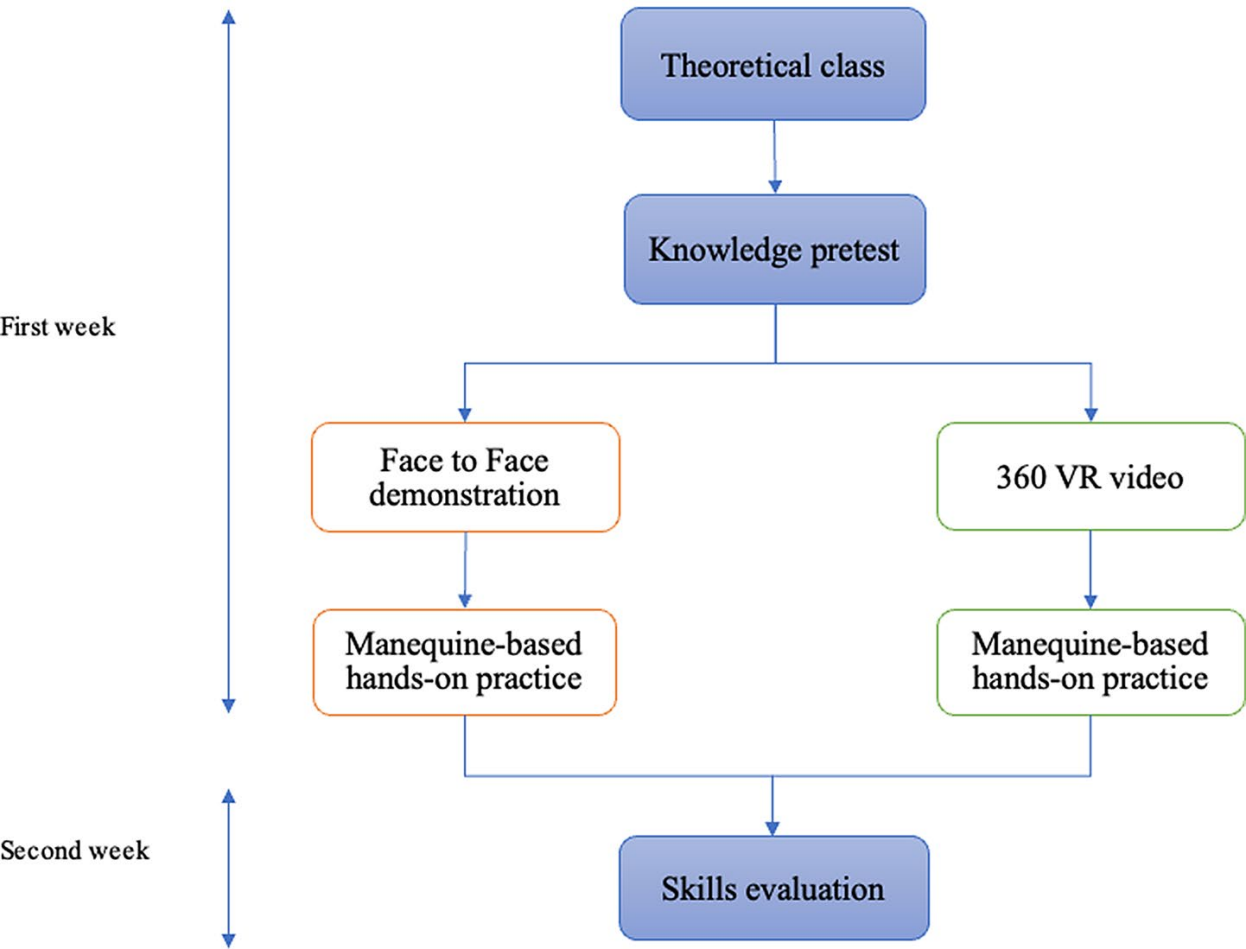
Study procedure

To assure equal distribution in terms of academic achievement or intelligence, the pretest score was used as a factor to block randomize the students into the 360º VR video training group (360º VR group: an intervention group) and the face-to-face traditional training group (face-to-face group: a control group). A block size of 2 resulted in 31 blocks, and students assigned from each block into the face-to-face and 360º VR groups. Figure 1 shows the study procedure during the course.

### Development of the 360º VR video

**Fig. 2:**
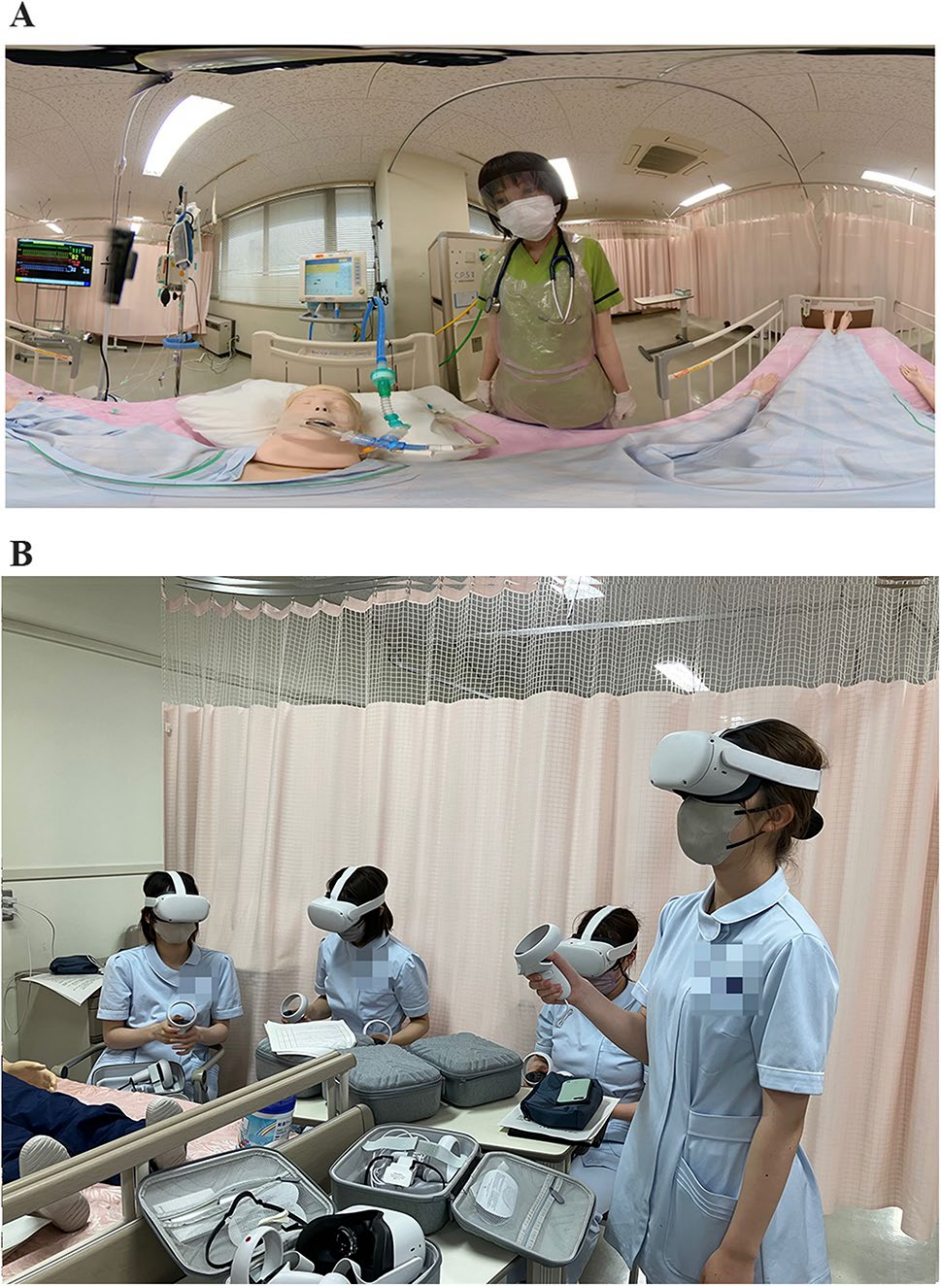
A shot from the 360º VR video. A shot of nursing students watching the procedure with HMDs

A video of a certified nurse in critical care performing closed-suction procedure in a high-fidelity mannequin-based simulation was recorded with Insta360 (ONE X2). The procedure of suctioning was conducted in a step-by-step manner following a checklist developed by the research team. The video involved a voice over of the instructor explaining the procedure, and the nurse performing the procedure. This was edited using Adobe Premiere Pro ver (23.2.0); the final product of the video was 18 min divided into three phases for better understanding of the procedure: Preparation and assessment phase; instrument identification and oral suction phase; tracheal suction and patient report phase (Fig. 2).

### 360º VR group

After the theoretical class, the 360º VR group watched (see Fig. 2a) the 360º VR video using the HMD of Meta Quest 2 individually; after then, the students answered the VR safety questionnaire developed by the researcher. For hands-on practice, the students engaged in self-directed practice with group feedback in 5 subgroups for 90 min; each subgroup containing 6 students except one with 7 students.

### Face-to-face group

The face-to-face group attended the face-to-face demonstration of the same nurse that carried out the procedure in the video using the researcher developed checklist. After then, the students engaged in hands-on practice in 5 subgroups for 90 min; each subgroup containing 6 students except one having 7 students; clinical instructors (experienced registered nurses) were present in each subgroup during the practice.

For both groups, the critical care nurse captured in the video was available between the intervention and control groups to address the students’ concerns and questions. After the hands-on practice, the control group was provided the usual supplemental procedural video for closed suctioning attached to their study material, and the intervention group could either rewatch the 360º VR video with VideoLAN Client (VLC) player or request for use of the HMD at their convenience.

A week after the skill demonstration and hands-on practice, a total of 9 instructors (nursing faculties and registered nurses) assessed and evaluated the students’ closed suction skills (including oral suction) using the procedure checklist. To ensure consistent evaluation, a session was held to communicate the grading criteria. The students were requested to perform the procedure in an Objective Structured Clinical Exam (OSCE) and evaluated by clinical instructors. At the end of the skills evaluation, both groups answered the Microsoft Forms on knowledge, satisfaction, and confidence. In addition, the 360º VR group answered the VR perception questionnaire. According to the study procedure, it was planned to explore the perception of the face-to-face group on VR by watching the 360º VR video after skills evaluation, but none of the students watched the 360º VR video.

### Evaluation outcomes and instrument

Evaluation was conducted under the framework of psychomotor skills, knowledge, confidence, and satisfaction of the closed tracheal suction technique. For the 360º VR group, perception and VR sickness symptoms were also explored.

### Closed tracheal suction checklist (including oral suction)

A Closed tracheal suction (including oral suction) checklist was developed from available literature review of evidence-based practice [31–37] to evaluate the skills of the nursing students (supplementary file 1). To ensure the validity of the checklist, the developed checklist was submitted to certified critical care nurses of Hiroshima University Hospital, and a version of procedure checklist available at the unit was received by the researchers as a guide. The checklist was further modified resulting in 38-steps procedure (items) checklist. To assign grades to the steps, each step was dichotomized to critical and non-critical. For a critical step, a score of 4, 2, 0 was assigned to satisfactory, unsatisfactory, and not performed, respectively; a score of 2, 1, 0 was assigned to a non-critical item as aforementioned for the level of performance. The criterion for the three level of performance was outlined for each item for consistent rating. To establish the content validity, using Lynn’s (1986) technique [38], the checklist was submitted to four certified critical care nurses; the relevance, accuracy of terminology, and grading of the steps were evaluated. The checklist was reviewed and modified based on the experts’ opinion, and the final checklist score ranges from 0 to 64. The item-level content validity index (I-CVI) was computed for each item; the scale-level content validity index of universal agreement (S-CVI/UA) was 0.97.

### Knowledge test scores for suction in ventilated patients

The researchers developed practical knowledge questions on tracheal suctioning (supplementary file 2). A total of 24 questions were outlined, and after the researchers’ group discussion, it was reduced to 17 questions. This was pretested with two certified critical care nurses for an expert-driven pretest to assess the face and construct validity of the questionnaire. The nurses answered the questionnaire, and suggested modifications or discard of some questions were addressed accordingly. After then, two questions were added, and 19 questions were pretested with two different certified critical care nurses. In order to achieve a 20-questions questionnaire, one question was included to the final expert-driven pretest. From the 20 questions developed, 7 questions, which were identified to address the basics and overview of tracheal suctioning, were used for pretest. For the post test, the total of 20 questions was administered; the correct answer is given 1 point, and the incorrect answer is given 0 points.

### Degree of satisfaction and confidence in learning

To assess the students’ satisfaction and confidence, the Japanese version [39] of the Students Satisfaction and Self-Confidence in Learning by the National League for Nursing (NLN) was adopted. It consists of 13 questions in two different questionnaire; five questions for satisfaction and eight questions for self-confidence. The questionnaire is on a 5-point Likert scale form 1 = strongly disagree, 2 = disagree, 3 = undecided, 4 = agree, and 5 = strongly agree; the higher the score, the higher the satisfaction and confidence. The satisfaction score ranges from 5 points to 25 points and confidence from 8 points to 40 points. As reported by NLN, the Cronbach alpha for the satisfaction and self-confidence are 0.94 and 0.87, respectively. In this study, the Cronbach alpha for satisfaction and self-confidence is 0.93 and 0.92, respectively.

### Perception of 360º VR use (including open-ended questions)

The perception of 360º VR video group was assessed with an adapted tool from Peart et al [40] study. The tool was developed based on the Technology Acceptance Model (TAM) and included 6 items on a 7-point Likert scale (strongly disagree, disagree, somewhat disagree, cannot decide, somewhat agree, agree, and strongly agree) and 2 open ended questions. However, to fit in our study, only one of the open-ended questions was retained (is there a way that the use of X could be improved). The tool was forward and back translated by the researchers, and an additional two questions (1. How was the comfort and ease of understanding of VR; 2. If you notice anything else or have any impressions, please write it down) were added to the open-ended questions. In Peart et al [40], the Cronbach alpha was > 0.7. As the tool was translated and adapted, the Cronbach alpha in this study is 0.61.

The safety questionnaire was developed to explore the side effects of using the VR. The VR sickness symptoms explored in the questionnaire were based on the Meta Quest 2 health and safety manual and other VR studies [19, 25, 41, 42]. It consisted of two questions (1) did you have any symptoms (2) please, pick all that applies. Ten symptoms were provided as option with an “other” option to allow for free answer.

### Ethical consideration

The study was conducted according to the Declaration of Helsinki and the Ethical Guidelines on Clinical Studies of the Ministry of Health, Labour and Welfare of Japan. This study was reviewed and approved by the Hiroshima University Epidemiological Ethics Review Committee (E2023-0054). One of the researchers who was not part of the adult health nursing course explained the study purpose and data collection procedure, and consent was received from students agreeing to secondary use of the data. It was explained that not consenting to the provision of data obtained in class would not affect the class grade in any way, and there would be no disadvantage on the part of the students; a written informed consent was obtained from all the students. Therefore, to ensure the class instructors would not be able to identify which students had consented, consent procedure and data extraction were done by the research coordinator.

### Data analysis

#### Skills checklist and questionnaires

Data analysis was performed with JMP, Pro 17 (SAS Institute Inc., Cary, NC, 1989–2023). Due to the non-normal distribution, descriptive data are presented in median, quartile, frequency, and percentage. Wilcoxon rank-sum test was used to compare the two groups in skills, knowledge, satisfaction, and confidence. The perception is presented as frequency, and percentages based on the level of agreement on the Likert scale; the open-ended questions were analyzed following the conceptual content analysis method to describe the attitudinal and behavioral responses of the students toward the 360º VR video. The VR side effects are presented as frequency and percentage. The level of significance was considered at 0.05.

#### *Open-ended questions for feedback*

The open-ended questions were analyzed following the conceptual content analysis method to describe the attitudinal and behavioral responses [43] of the students toward the 360º VR video. The coding unit of analysis was defined as the individual theme; according to Minichiello et al. as cited in Zhang and Wildemuth [44], this strategy is to capture the expressions of an idea. In the initial stage of the open coding, the phrases used by the students were singled out to enable in vivo codes; this prevents contamination of the data and allows valid representation of the students’ idea [45]. Furthermore, the in vivo codes of similar ideas were grouped together; the codes were organized to derive categories. The process of the analysis was examined by the research team and disagreements in the process were addressed accordingly.

## Result

**Fig. 3:**
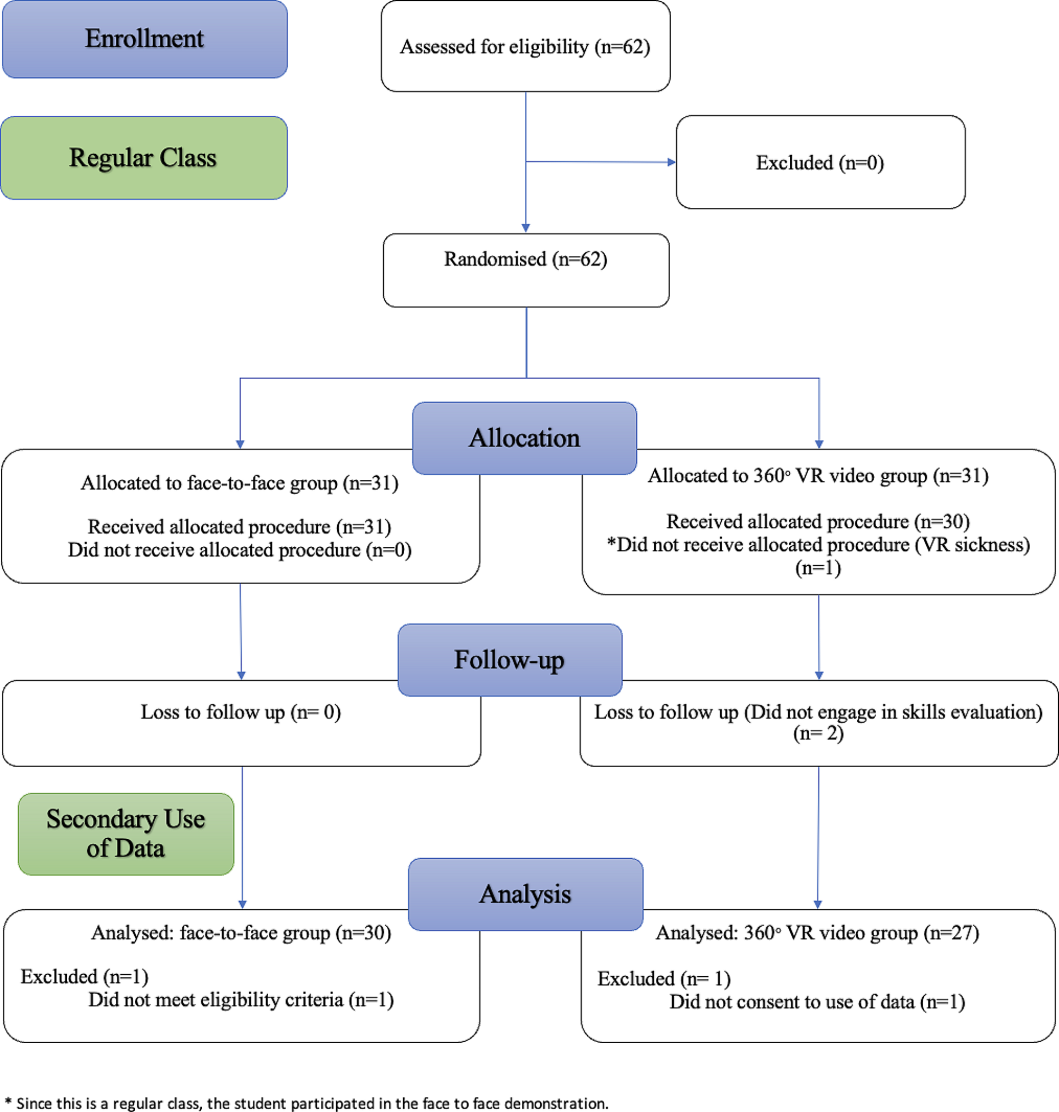
CONSORT flow chart

Out of the 62 students randomized for the study, data of 57 students were used for analysis (Fig. 3). They were all females and aged between 20 and 22 years.

Table 1 shows the median score of both groups based on the knowledge pretest for randomization. Both groups were equally randomized, and there is no significant difference between the two groups.

**Table 1:** Pretest score for students allocated into face-to-face and 360º VR Video groups

								(*N* = 57)
Teaching method	n	Median	25% Quartile	75% Quartile	Max	Min	Z	*P*-value
Face-to-Face	30	5	4	6	7	3	0.158	0.874
360º VR video	27	5	5	6	7	3		

Max = maximum score (7)

Min = minimum score (0)

There is no significant difference between the two groups based on the pretest used for block randomization

Scores based on knowledge pretest

### Psychomotor skills of the students on closed endotracheal suctioning

**Fig. 4:**
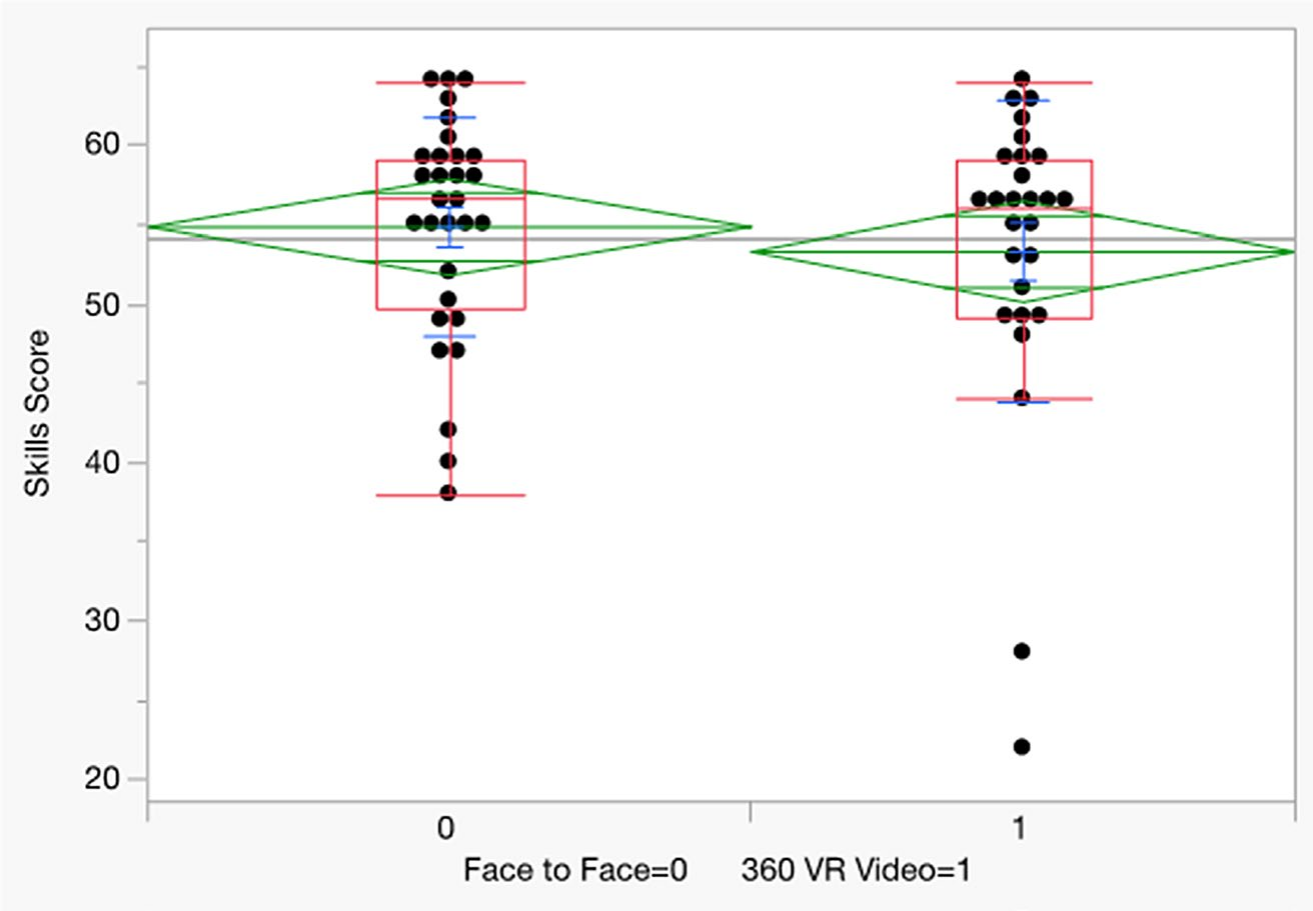
Psychomotor skill scores of closed tracheal suction (including oral suction)

The median scores for face-to-face and 360º VR groups were 56.5 and 56.0, respectively; there is no significant difference between the two teaching methods (Z= -0.385, *P* = 0.700) (Table 2; Fig. 4).

**Table 2:** Psychomotor skills score of closed tracheal suction (including oral suction)

								(*N* = 57)
Teaching method	N	Median	25% Quartile	75% Quartile	Max	Min	Z	*P*-value
Face-to-Face	30	56.5	49.75	59	64	38	−0.385	0.7
360º VR video	27	56.0	49	59	64	22		

Max = maximum score (64)

Min = minimum score (0)

There is no significant difference between the two groups

Difference was evaluated using Wilcoxon 2- sample (Rank Sum)

### Knowledge of the nursing students

Among the 57 students, 55 students answered the knowledge test (Face-to-Face = 29; 360º VR = 26). The median scores for face-to-face and 360º VR groups were 16 and 15, respectively. No statistically significant difference was observed between the groups (Z = 0.059, *P* = 0.952). Table 3 presents the median scores of the respective teaching methods.

**Table 3:** Knowledge scores of closed tracheal suction (including oral suction)

								(*N* = 55)
Teaching method	N	Median	25% Quartile	75% Quartile	Max	Min	Z	*P*-value
Face-to-Face	29	16	14	16.5	19	9	−0.059	0.952
360º VR video	26	15	13	17	19	10		

Max = maximum score (20)

Min = minimum score (0)

There is no significant difference between the two groups

Difference was evaluated using Wilcoxon 2- sample (Rank Sum)

### The levels of satisfaction and confidence

**Fig. 5:**
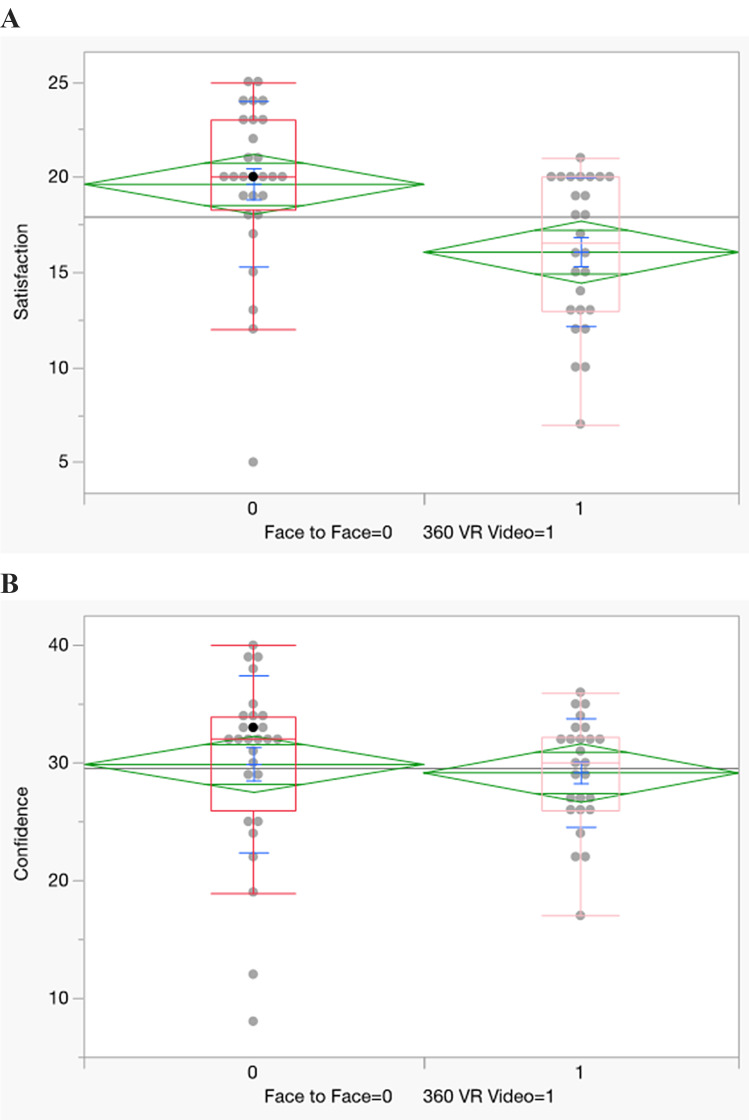
Satisfaction scores of students using the face-to-face and 360º VR Video teaching method. Confidence scores of students using the face-to-face and 360º VR Video teaching method

Tables 4, 5 and 6; Fig. 5 presents the comparison between the level of satisfaction and confidence of the face-to-face and 360º VR groups. While there was statistically significant difference in the level of satisfaction between the two groups, the difference in the confidence level was not significant. On the item-level satisfaction, all the statements are statistically significant except, “the teaching material used in this simulation were motivating and helped me to learn” (*P* = 0.063).

**Table 4:** Satisfaction and Confidence scores of students using the face-to-face and 360º VR Video teaching method

					(*N* = 54)
Variables	Teaching method	N	Median	Z	*P*-value
Satisfaction	Face-to-Face	28	20	-3.252	0.001
	360º VR video	26	16.5		
Confidence	Face-to-Face	28	32	-1.097	0.273
	360º VR video	26	30		

Satisfaction: maximum score (25)

minimum score (5)

Confidence: maximum score (40)

minimum score (8)

Difference was evaluated using Wilcoxon 2- sample (Rank Sum)

Scores based on a five-point Likert scale

**Table 5:** Satisfaction scores of students using the face-to-face and 360º VR Video teaching methods

Satisfaction	Teaching method	N	Median	Z	*N* = 54 P-value
The teaching method was helpful and effective.	Face-to-Face	28	4	−2.877	0.004
	360º VR video	26	3		
The simulation provided me with a variety of learning materials and activities to promote my learning	Face-to-Face	28	4	−2.899	0.004
	360º VR video	26	4		
I enjoyed how my instructor taught the simulation.	Face-to-Face	28	4	−3.194	0.001
	360º VR video	26	3.5		
The teaching materials were motivating and helped me to learn.	Face-to-Face	28	4	−1.863	0.063
	360º VR video	26	4		
The way my instructor(s) taught the simulation was suitable to the way I learn.	Face-to-Face	28	4	−3.674	< 0.001
	360º VR video	26	3		

Satisfaction: maximum score (25), minimum score (5)

Difference was evaluated using Wilcoxon 2- sample (Rank Sum)

Scores based on a five-point Likert scale; higher score indicating better satisfaction

**Table 6:** Confidence scores of students using the face-to-face and 360º VR Video teaching methods

Confidence	Teaching method	N	Median	Z	*N* = 54 P-value
I am confident that I am mastering the simulation activity my instructors presented to me.	Face-to-Face	28	4	−1.811	0.07
	360º VR video	26	2		
I am confident that this simulation covered critical content	Face-to-Face	28	4	0.359	0.719
	360º VR video	26	4		
I am confident in my skills development and obtaining the required knowledge to perform necessary tasks in a clinical setting	Face-to-Face	28	4	−0.702	0.483
	360º VR video	26	4		
My instructors used helpful resources to teach the simulation.	Face-to-Face	28	4	−0.918	0.359
	360º VR video	26	4		
It is my responsibility to learn what I need to know from this simulation activity.	Face-to-Face	28	4	0	1.000
	360º VR video	26	4		
I know how to get help on the concepts covered in the simulation.	Face-to-Face	28	4	−0.710	0.476
	360º VR video	26	4		
I know how to use simulation activities to learn critical aspects of these skills.	Face-to-Face	28	4	0.020	0.984
	360º VR video	26	4		
It is the instructor’s responsibility to tell me what I need to learn of the simulation activity content during class time.	Face-to-Face	28	4	0.647	0.518
	360º VR video	26	4		

Satisfaction: maximum score (40), minimum score (8)

Difference was evaluated using Wilcoxon 2- sample (Rank Sum)

Scores based on a five-point Likert scale; higher score indicating better confidence

### The perception of the 360º VR use

Table 7 shows the perception of the 360º VR group regarding the VR use. It was intended to introduce the 360º VR to the face-to-face group and explore their perception, but no student in the face-to-face group volunteered to watch 360º VR video after the class. Therefore, the endpoint of comparing how both groups perceived the 360º VR video use was not achieved. Out of the 27 students in the VR video group, a total of 26 students answered the questionnaire. Among them, 81% of the students disagreed that VR is a bad idea, and 42.3% perceived the technology useful for learning. About 62% agreed that VR makes learning more interesting; however, 35% of the student cannot decide if they would like to use VR video in future clinical skills, and 42% disagreed.

**Table 7:** Perception of students using the 360º VR Video

Question	Strongly disagree	Disagree	Somewhat disagree	Cannot decide	Somewhat agree	Agree	N (%) Strongly agree
I find VR useful for learning	0 (0)	5 (19.2)	4 (15.4)	6 (23.1)	7 (26.9)	4 (15.4)	0 (0)
VR helped me develop confidence in performing the skill	0 (0)	5 (19.2)	7 (26.9)	7 (26.9)	5 (19.2)	2 (7.7)	0 (0)
I find VR easy to use	0 (0)	10 (38.5)	8 (30.8)	5 (19.2)	3 (11.5)	0 (0)	0 (0)
Using VR is a bad idea	7 (26.9)	9 (34.6)	5 (19.2)	3 (11.5)	1 (3.9)	1 (3.9)	0 (0)
VR makes learning more interesting	1 (3.9)	2 (7.7)	2 (7.7)	5 (19.2)	7 (26.9)	8 (30.8)	1 (3.9)
I would like to use VR in future clinical skills training	2 (7.7)	2 (7.7)	7 (26.9)	9 (34.6)	5 (19.2)	1 (3.9)	0 (0)

N (%): Number of participants and percentage; *N* = 26

### VR sickness symptoms

Table 8 presents the VR sickness symptoms reported by the students using 360º VR video. More than half of students (62.5%) experienced VR sickness symptoms, and “feeling of drunk” is the highest.

**Table 8:** VR sickness symptoms reported by the students using 360º VR Video

VR sickness symptoms	*N* = 24
	N(%)
	15 (62.5)
Specific symptoms	*N* = 24
	N(%)
Feeling of drunk	12 (50.0)
Vomitting	0 (0)
Nausea	1 (4.2)
Diziness	0 (0)
Wobble	1 (4.2)
Fall due to wobble	0 (0)
Sweating	0 (0)
Eye fatigue	8 (33.3)
Fatigue	1 (4.2)
Epilepsy	0 (0)
Headache	3 (12.5)
Neck pain	1 (4.2)
Face pain	1 (4.2)

N (%): Number of participants and percentage; *N* = 24

### Qualitative analysis of the open-ended questions

In order to supplement the quantitative data, the open-ended questions were analyzed, and yielded 5 categories (see Table 9). The students appreciated the ready to use, immersiveness and realism; however, symptoms and discomfort, burdensomeness and production limitations were improvements recommended. Moreover, the impression (see Table 10) of the students concerning the 360º VR video was preference for face-to-face teaching because it offers the opportunity to engage with the instructor and ask practical questions. Another aspect is some students believed regular videos are easier to watch. One of the students noted “Difficult, because I have to use equipment (headset) to review videos”.

**Table 9:** Qualitative analysis of students’ comments on 360º VR video teaching method (*N* = 26)

Categories	Positives/Improvements
*Immersiveness and Realism*	***(Positive)*** - The immersiveness and close to reality of the demonstration was appreciated by the students. A total of 11 students believed it was very realistic and this could be leveraged as an alternative to face-to-face demonstration. The students represented this with positive reviews like “up close and personal” “sense of realism” “immersive experience is high”. ***Direct Quotes***: “It was nice to have a sense of realism.” “The head set was heavy. But it was very realistic. It was more up close and personal than actually seeing a faculty member’s demonstration.” “In person, it was difficult to see the procedure of the teacher at the front of the classroom in a large group, but in VR it was easier to see the procedure up close.”
*Ready to Use and Pacing*	***(Positive)*** - The teaching method offers the opportunity to reuse the video to review procedures at convenience. Four students considered the reusability as an effective strategy to teach procedures. This appeared in the texts as “review exercises” and “watch over and over”. ***Direct Quotes***: “It was very realistic. I liked the fact that I could go back and look at the parts I didn’t understand over and over again.” “I think if you think about when to use VR, it can be used effectively. (e.g. reviewing exercises in VR at home)” “I wasn’t sure if it needed to be in VR. But I do think being able to watch it over and over again is effective for learning.”
*Production Limitation*	***(Improvement)*** - The technical issue and limitations associated with the production like inability to look down, audiovisual problem, and uncomfortable positioning were some of the concerns of the students. Students (*N* = 13) represented this as but not limited to “hard to hear distant sounds”, “angle above patient”, and “difficult to look down”. ***Direct Quotes***: “Because the angle was from above the patient, I had to look all the way down to the left to see the nurse’s procedure, which was a little uncomfortable. Also, the saturation monitor was behind the patient, so I had to turn around to see it.” “I thought it would be better if the angle could be changed to a position that is easier to see. It would be better if the video could be viewed from the perspective of the person actually performing the procedure, so that the understanding could be deepened.” “It would be easier to understand if the camera position could be set at the same height as when it is implemented. It would be easier to review if you could make the rewind interval a little shorter, say 5 s.”
*Symptoms and Discomfort*	***(Improvement)*** - The VR sickness symptoms and discomfort experienced during the demonstration was reported by the students. Students (*N* = 15) recounted this as but not limited to “easily intoxicated”, “a bit sick”, “head is heavy”, and “eye strain and headaches”. ***Direct Quotes***: “I feel that I was a little easily intoxicated. It was easy to feel a bit sick.” “It was immersive and realistic, and I could feel what was going on firsthand, but the distortion of the screen and the eye strain and headaches associated with it made it difficult for me to use it on a daily basis” “The camera’s viewpoint was directly above the patient, so it was easy to see the procedure, but it was difficult to turn around between the doctor and the monitor. The head set was heavy, so there was a burden on the face, and I felt sick”
*Burdensomeness*	***(Improvement)*** - The difficulty in navigation through the activity-oriented demonstration was a source of concern to the students. Students (*N* = 9) expressed this as phrases like “little uncomfortable”, “difficult perspective”, “had to turn around”, “hassle to put it on”, and “image too large”. ***Direct Quotes***: “I need to turn my head around and I’m tired, so I think it would be good to improve that” “I had to move my head to see some parts of the procedure, so it was more difficult to understand than a live lecture” “It was a hassle to have to put it on and watch the video every time there was a question” “It was hard to tell where things were”

**Table 10:** Nursing students’ impression of 360º VR video teaching method (*N* = 26)

Impressions	
	The students were asked of their impression of the new technology, and the overriding concern was for the use of regular video and face-to-face teaching. This was evident is some of the students’ statements. ***Direct Quotes***: “It was difficult to watch and study VR over and over again, so it was easier to review if I could watch regular videos together. It was good that there was a sense of realism.” “I thought that we cannot ask the instructor to ask what we did not understand straight away.” “I thought a demonstration would be better, because I could ask any questions I had at the time on the spot.” “I appreciate the ability to replay videos when I want to review, but on a computer monitor, regular videos are easier to watch.” “Compared to watching videos on a PC, the sense of presence is superior, but considering the hassle of wearing the device, eye strain and headaches after watching, daily use or use for more than 20 ~ 30 minutes is not feasible.”

## Discussion

This study assesses the effectiveness of 360º VR video in teaching nursing procedure over the traditional face-to-face teaching method. Our hypotheses were that the 360º VR video group would demonstrate better skill, knowledge, confidence, and satisfaction level than the face-to-face group; these hypotheses were not supported as there was no statistically significant difference between the groups in skill, knowledge, and confidence, and the face-to-face group had higher satisfaction level than the 360º VR video group. On the other hand, the qualitative result suggested that VR symptoms, burdensomeness, and production limitation, were negative experiences often cited; the feeling of immersion and the opportunity to reuse the video were positive aspects of the 360º VR video perceived by the students.

The research on 360º VR video is a relatively new area in medical education, and teaching method for comparison are not usually the same, which makes the evidence disparate. A complementary study by Arents et al [41] compared a group of students learning medical obstetrics and cesarean section in face-to-face combined with 360º VR video to the face-to-face group only; there was no statistically significant difference between the groups in knowledge retention. Similar to our findings, in Sweden, Ulrich et al [46] in a three-arm study (i.e., 360º VR video group, Regular video group, Traditional teaching group) compared physiotherapy students on academic performance and students’ learning satisfaction. The three methods have the same effect on enhancement of academic performance; however, the traditional teaching was more effective than both 360º VR video and regular video in students’ learning satisfaction. In a similar approach to our study in Saudi Arabia, Sultana et al [47] compared 360º VR group of medical students learning communication skills with a conventional group that received interactive lecture on the same skills. Contrary to our findings, the 360º VR video group scored significantly higher than the conventional group in Multiple Choice Questions (MCQs) and OSCE. In other studies comparing 360º VR video to 2D, Yoganathan et al [48] compared first year postgraduate doctors’ skills of knot tying using 360º VR video and 2D video. The 360º VR video arm performed significantly better than the 2D arm. In Taiwan, Chao et al [25] compared nursing students learning nasogastric tube feeding with 360º VR video and regular demonstration video on the outcomes of skills, knowledge, satisfaction, and confidence. There was no statistically significant difference between the two groups in skill, knowledge, and confidence; however, the VR group demonstrated higher satisfaction than the traditional video group.

It was expected that the immersiveness, higher engagement and enthusiasm associated with the use of 360º VR video could afford the students a higher possibility of effective learning [49–52]. The non-significant study outcomes might be that the action-oriented activities involved cognitively demanding details that requires extra attention; highly complex learning environments increase the cognitive load [53]. Based on the students’ feedback, there were concerns on having to move and turn in the virtual space to follow up on the procedure; it is the first time to use such novel technology to learn procedure, and it might be tiring and distracting. One of the students stated that “I had to move my head to see some parts of the procedure, so it was more difficult to understand than a live lecture”; another student noted “the saturation monitor was behind the patient, so I had to turn around to see it.” This could possibly be addressed by providing orientation on what to expect in the virtual space. Moro et al [52] maintained that there is a risk of distraction with the use of VR technology; the participants reported spending more time on exploring the technology rather than learning the contents. Chao et al [25] also maintained similar conclusion. Likewise, the experience of VR symptoms is a convergent finding as it is supported by the quantitative and qualitative data; this could have hampered the learning experience. The result of the VR symptoms revealed that, about 63% (15 out of 24 students) of the students reported VR related symptoms in this study. Moro et al [52] reported that the adverse effects of dizziness, blurred vision, and headaches were felt by 40%, 35%, and 25% of the students, respectively; this could have an impact on learning quality. In this study, one of the students states “It was immersive and realistic, and I could feel what was going on firsthand, but the distortion of the screen and the eye strain and headaches associated with it made it difficult for me to use it on a daily basis”. The participants in Van De Broeck [54] concluded that although the immersiveness with HMDs offer the best user experience, they are associated with cognitive burden, motion sickness and physical discomfort. Somrak et al [55] reported negative association between VR sickness discomfort levels and user experience.

The satisfaction level of the face-to-face group was significantly higher than the 360º VR video group in this study. However, considering the statement on the satisfaction questionnaire, which states, “the teaching materials were motivating and helped me to learn”; there was no statistically significant difference between the scores of the 360º VR video and face-to-face group. This means that both groups equally agreed on the teaching methods being motivating and helpful. For the overall satisfaction, to begin with, the possible reason might be that this study explores the satisfaction of a video-based group, which has been believed by students to lack the opportunity to ask questions and interact with the instructor [56]. Likewise, the teaching method was adopted as an alternate approach rather than blending face-to-face with video for the intervention group. Our qualitative finding suggests that the 360º VR video group prefers the presence of the instructor in face-to-face teaching. Similar to this findings, Alqahtani et al [28] concluded that students were reluctant to replace live demonstration with procedural video; only 40% of the students preferred the procedural video compared to the 59% in face-to-face demonstration. Also, another statement on the satisfaction questionnaire, which states “The way my instructor(s) taught the simulation was suitable to the way I learn”; the face-to-face group scored significantly higher. Our students might have appreciated the interaction opportunity in the face-to-face teaching; the use of VR is a new method of teaching and students are already familiar and accustomed to the face-to-face teaching method. This study compares 360º VR video to face-to-face teaching; It is worth pointing out that the studies demonstrating significantly higher satisfaction for 360º VR video compared two different video-based method (VR versus regular demonstration video). It is difficult to extrapolate these studies' result on satisfaction to our study based on the difference in approach. A three-arm study by Ulrich et al [46] found that traditional teaching was more effective than 360º video and regular video in students’ learning satisfaction. Additionally, the discomfort experienced through the VR sickness symptoms might have affected their satisfaction level. For perception, only a moderate percentage (42.3%) of the 360º VR video group students rated VR useful for learning, and 23% would like to use it in future skills training. Contrarily, in Arents et al [41] a complementary approach, 100% of the students rated it useful, and 83.4% reported that more 360º VR videos should be used in future courses.

### Limitations and strengths

The limitation in this study is, to begin with, the intervention was open to the student, and this could have alerted and give the impression of non-conformity to the new technology as a replacement for the established teaching method. This effect and performance bias could have been mitigated with a blinded study, but blinding was not feasible. Moreover, the 360º VR video was only content validated; the production was not validated for use. This could have also affected the students experience of the technology leading to production limitation. Additionally, all the students in our study were females; if the genders were mixed, there could be more generalization as gender factors in the acceptance of technology. Subsequently, our sample was a convenience sample of the nursing students enrolled in the course; a larger sample size could have achieved a normal distribution and mitigate the effect of using a non-parametric test. Also, this is a single-center study in Japan; the findings cannot be generalized to other nursing students.

## Conclusion

Although face-to-face demonstration is the established method of teaching psychomotor skills to nursing students, the use of 360º VR video could achieve similar learning effect as an alternative approach. Nevertheless, only a moderate percentage of the students in the 360º VR video group perceived the technology useful for learning. While this is true for learners’ performance, there is need for more studies to explore the students’ satisfaction when used as an alternative. Moreover, factors like ease of use and VR sickness symptoms experienced by users hinder the acceptance of the teaching method.

### Electronic supplementary material

Below is the link to the electronic supplementary material.

Supplementary Material 1

Supplementary Material 2

## Data Availability

The datasets used and analyzed during the current study are available from the corresponding author on reasonable request.

## Abbreviations

CVI: Item-level content validity index
CVI/UA: Scale level content validity index of universal agreement
HMD: Head Mounted Display
ICU: Intensive Care Unit
MCQ: Multiple Choice Question
NLN: National League for Nursing
OSCE: Objective Structured Clinical Exam
TAM: Technology Acceptance Model
VR: Virtual Reality
VLC: VideoLAN Client
2D: Two-dimensional
