# Supplementary material for: Face-to-face versus 360° VR video: a comparative study of two teaching methods in nursing education
Source: BMC Nurs. 2024 Mar 25;23:199. doi: 10.1186/s12912-024-01866-4 (PMC10962166; doi:10.1186/s12912-024-01866-4)
Supplement: Supplementary file 1 — Supplementary Material 1 [file 12912_2024_1866_MOESM1_ESM.pdf]

Knowledge test

Answer the following about suctioning of patients on mechanical ventilator

1. All of these are indications for suctioning, except

A. Gradual or sudden decrease in oxygen saturation

B. Secretions in the artificial airway

C. Resonance sound on percussion

D. Rhonchi on auscultation

2. On oral suctioning, the nurse should ensure ALL of the following except

A. Move the catheter along the gum-line towards the pharynx in a circular motion

B. Suction gently and with caution

C. Insert the catheter deep to the end of the tongue to the end of the tongue

D. Observe color and amount of oral secretion

3. Which of these is correct for instrument needed for tracheal suctioning?

A. Suction device, connecting tube, closed suction tube, oral suction tube, rinse solution, alcohol swab

B. Suction device, connecting tube, nasogastric tube, rinse solution, alcohol swab

C. Suction device, connecting tube, closed suction tube, oral suction tube, rinse solution, venturi mask

D. Suction device, connecting tube, biteblock, oral suction tube, rinse solution, alcohol swab, gloves

4. Routine instillation of 0.9% sodium chloride to loosen the secretion is recommended before suctioning

A. Yes

B. No

5. During superficial airway suctioning, the catheter should be introduced to a depth more than the artificial airway

A. Yes

B. No

6. Introducing the catheter more than the artificial airway can cause the following, except

A. Pneumonia
B. Mucosal trauma
C. Bleeding

7. Suction pressure 30 kPa over may increase tracheal mucosal damage

A. Yes
B. No

8. How often should patient be suctioned

A. Routinely
B. When physical findings support the need

9. The following are complications of tracheal suctioning

A. Bronchospasm
B. Atelectasis
C. Hypertension or hypotension
D. Arrythmias
E. All of the above

10. Tracheal mucosal damage may be prevented through all, except

A. Choosing the appropriately sized catheter
B. Maintain proper suction technique
C. Maintain adequate suction pressure
D. Administration of 100% of oxygen before suctioning

11. A change in the airway secretion is suggestive of

A. Hypoxemia
B. Infection
C. Hypercapnia
D. Pneumothorax

12. High cuff pressure may cause

A. Tracheal mucosa necrosis

B. Air leakage

C. Infection

D. Atelectasis

13. In closed suction technique, the patient is disconnected from the ventilator to allow passage of catheter

A. Yes

B. No

14. Suction should be applied while inserting the in-line catheter into the artificial airway

A. Yes

B. No

15. The number of suction passes should be based on the amount of secretions and the patient’s clinical assessment

A. Yes

B. No

16. The right size of the suctioning tube should be approximately the half diameter of the tracheal tube.

A. Yes

B. No

17. What is the right cuff pressure? Write down the number and unit in the bracket.

( )

18. Under-inflation of the endotracheal cuff is associated with which of the following?

1. Atelectasis
2. Tracheal mucosal necrosis
3. Ventilator associated pneumonia
4. Bronchiectasis

19. Tracheal suction should be performed at a suction pressure of (   ) kPa or less. Write the correct value in ( ).

(   )

20. To minimize decrease in oxygen saturation, the suction time when applying suction pressure should be within (   ) seconds.

(   )
