# Supplementary material for: Face-to-face versus 360° VR video: a comparative study of two teaching methods in nursing education
Source: BMC Nurs. 2024 Mar 25;23:199. doi: 10.1186/s12912-024-01866-4 (PMC10962166; doi:10.1186/s12912-024-01866-4)
Supplement: Supplementary file 2 — Supplementary Material 2 [file 12912_2024_1866_MOESM2_ESM.pdf]

Closed tracheal suction checklist (including oral suction)

	Step	S	U	NP
1	**Implementation of the universal precaution: Disinfect hands, wear gloves and appropriate PPE**			
	1. Disinfect hand by alcohol gel	2	1	0
	2. Wear apron	1		0
	3. Wear face mask	1		0
	4. Put on face shiled or googles	1		0
	5. Put on gloves	1		0
2	Identify the patient	2		0
3	**Assess for need for tracheal suctioning (Perform visual, palpation, and auscultation)**			
	1. Assess for cyanosis or pallor by checking the skin color, and visual inspection of respiratory parameters (e.g., respiratory pattern and efforts, intercostal and suprasternal retraction, shallow respiration)	2	1	0
	2. Auscultate the lungs' field with the use of stethoscope diagphram in a sytemic pattern and identified the lung sound	4	2	0
	3. Check the bedside monitor to check for deviations in the respiratory and hemodynamic parameters	2	1	0
	4. Check the Ventilator monitor for deviations suggestive of inadequate oxygenation or accumulation of secretion (e.g., sawtooth pattern, increased peak airway pressure)	Not evaluated		
4	Place the patient in appropriate position based on the disease condition and secretion location	Not evaluated		
5	Identify supplies needed: Suction device, connecting tube, closed suction tube, oral suction tube, rinse solution, alcohol swab, gloves, cuff manometer.	2	1	0
6	Explain to the patient the purpose and method of tracheal suctioning and obtain consent<br>Example: I heard sound of secretion, may I remove that from your mouth and tube?	2		0
7	Check the fixation status and depth of the tracheal tube	2	1	0
8	Check the insertion length of the closed suction tube	4	2	0
9	Check for proper cuff pressure by connecting the pilot ballon to a cuff manometer	2	1	0
10	Connect the connecting tube to the suction device			
11	Apply pressure to allow use of the suction device			
12	Adjust suction pressure to about 20kpa or 150mmHg and check for negative pressure by occluding the end of the connecting tube	4	2	0
13	Connect the oral suction tube to the connecting tube			
14	Aspirate oral secretions	4	2	0
15	Observe the color, properties, and amount of oral secretions			
16	Discard the oral suction tube and gloves by Glove-in-Glove or Bird-Beak technique	2	1	0
17	Disinfect hands and put on gloves			
18	Connect the suction tube above the cuff to the connecting tube			
19	Aspirate upper cuff secretions	2		0
20	Observe the color, consistency, and volume of the upper cuff secretions			

21	(If necessary) Operate ventilator and administer high-concentration oxygen<br>*Preoxygenate with 100% for 30-60 seconds	Not evaluated		
22	Remove the cap on the suction port of the closed suction tube and connect the connecting tube	2	/	0
23	Unlock the control valve			
24	Grasp the connection between the artificial airway and the closed suction tube with the non-dominant hand			
25	With the dominant thumb and forefinger, insert the closed suction tube into the artificial airway	2	1	0
26	With the dominant thumb, depress the control valve while withdrawing the suction tube	4	2	0
27	Observe the color, consistency, and volume of secretions, as well as respiratory and circulatory dynamics, while suctioning	2	1	0
28	Ensure that the closed suction tube is completely pulled up (Stopped the withdrawal when the black marker ring on the suction tube appears inside the sleeve at the proximal end (Visually check the line of the tube), then, release the control valve)	2	1	0
29	Repeat suctioning technique if unable to suction at one time (24-28) (Ensured recovery time between each pass)	Not evaluated		
30	Attach the rinse solution to the flush port and depress the contol valve to clean the inside of the tubing	2	1	0
31	Lock the control valve	2	/	0
32	Disconnect the closed suction tube from the connecting tube, clean the connector (suction port) of the closed suction tube with alcohol swab and put on the cap	2	1	0
33	Turn off the suction device	2	/	0
34	Remove gloves by Glove-in-Glove or Bird-Beak technique and sanitize hands	2	1	0
35	Remove apron and eye guard, and mask (in that order) and perform hand sanitization			
36	Check for changes in the patient's condition and synchronization with the ventilator	Not evaluated		
37	Inform the patient that the procedure has been completed	2	/	0
38	Record and report the procedure to an evaluator	2	/	0

S: The step was performed correctly

U: The step was underperformed

NP: The step was not performed

Note: / is not applicable, not evaluated (not evaluated in this study)
